# “Alphabet” Selenoproteins: Implications in Pathology

**DOI:** 10.3390/ijms242015344

**Published:** 2023-10-19

**Authors:** Carmen Beatrice Dogaru, Carmen Duță, Corina Muscurel, Irina Stoian

**Affiliations:** Department of Biochemistry, Carol Davila University of Medicine and Pharmacy, 050474 Bucharest, Romaniairina.stoian@umfcd.ro (I.S.)

**Keywords:** selenium, selenoproteins, cancer, type 2 diabetes, neurodegeneration

## Abstract

Selenoproteins are a group of proteins containing selenium in the form of selenocysteine (Sec, U) as the 21st amino acid coded in the genetic code. Their synthesis depends on dietary selenium uptake and a common set of cofactors. Selenoproteins accomplish diverse roles in the body and cell processes by acting, for example, as antioxidants, modulators of the immune function, and detoxification agents for heavy metals, other xenobiotics, and key compounds in thyroid hormone metabolism. Although the functions of all this protein family are still unknown, several disorders in their structure, activity, or expression have been described by researchers. They concluded that selenium or cofactors deficiency, on the one hand, or the polymorphism in selenoproteins genes and synthesis, on the other hand, are involved in a large variety of pathological conditions, including type 2 diabetes, cardiovascular, muscular, oncological, hepatic, endocrine, immuno-inflammatory, and neurodegenerative diseases. This review focuses on the specific roles of selenoproteins named after letters of the alphabet in medicine, which are less known than the rest, regarding their implications in the pathological processes of several prevalent diseases and disease prevention.

## 1. Introduction

It is now very well known that the history and importance of the implications of selenoproteins in health and diseases began in 1817 when the trace element selenium (Se) was first discovered by the Swedish chemist Jöns Jacob Berzelius and named after the Greek goddess of the Moon, Selene, and originally considered a naturally occurring toxicant. In 1957, this point of view changed thanks to Schwartz’s and Foltz’s unexpected discovery that selenium prevented liver necrosis in rats. This discovery changed the perception of selenium as a health threat. As time passed, selenium began to be viewed as an essential and beneficial trace element for health. Based on these discoveries, the era of selenoproteins started in 1974 when the American biochemist Thressa Campbell Stadtman added the famous and unique new amino acid selenocysteine (Sec, U) as the 21st “naturally occurring” in the genetic code [1]. Sec is cotranslationally inserted into nascent polypeptide chains in response to the UGA codon, known as the stop codon. For this “magic” to be possible, organisms evolved using the intensely researched insertion machinery requiring a cis-acting Sec insertion sequence (SECIS) element [2].

Regarding selenoproteins and selenoproteome, 25 selenoprotein genes corresponding to 25 selenoproteins have been identified in humans, showing different properties and functions, most broadly classified as antioxidant enzymes. This selenoproteome includes glutathione peroxidases (GPxs), iodothyronine deiodinases (DIOs), thioredoxin reductases (TRxRs), methionine sulfoxide reductases (Msrs), and selenoproteins named after letters of the alphabet (H, I, K, M, N, O, P, R, S, T, V, and W). GPxs are oxidoreductases that are involved in reducing varied hydroperoxides, such as hydrogen peroxide (H_2_O_2_) to H_2_O using glutathione (GSH). The DIO selenoproteins (DIO1, DIO2, and DIO3) are implicated in regulating thyroid hormones and catalyzing reductive deiodination. TrxRs are essential protein disulfide reductases in cells. Msrs are thiol (or selenol)-dependent oxidoreductases [2]. Several functions of the “alphabet” selenoproteins are listed in Table 1.

The functions of selenoproteins are altered in diseases associated with mutations of SECISBP2 (Selenocysteine Insertion Sequence-Binding Protein 2, SECIS Binding Protein 2) or SEPSECS (Sep (O-Phosphoserine) tRNA:Sec (Selenocysteine) tRNA Synthase) genes. SECISBP1 encoded a protein that represents an essential component of the machinery that carries out the insertion of Sec into selenoproteins. Some mutations generate only clinical phenotypes expressed in specific tissues due to the deficiency of particular selenoproteins, whereas other phenotypes have multifactorial causes [26]. All these patients have similarly low plasma selenium levels, reflecting low SELENOP and GPx3 synthesis and abnormal thyroid hormone values caused by the diminished activity of deiodinases. Their values are raised for FT4 and reverse T3 (rT3), normal to low for FT3, and normal to high for FSH [27,28,29]. Children have a growth slowdown, intellectual development delay, and motor coordination deficits [30,31]. Several patients have shown progressive muscular dystrophy in axial and proximal limb muscles that is very similar to the phenotype of the SELENON deficiency myopathy [32]. Another phenotype revealed azoospermia due to the compromised latter stages of spermatogenesis caused by a marked deficiency of testis-expressed selenoproteins GPx4, TXNRD3, and SELENOV [33,34,35,36,37]. Other phenotypes that include increased subcutaneous fat mass, insulin sensitivity, and cutaneous photosensitivity have possible multifactorial origins involving impaired antioxidant and ER stress defense [29]. Studies on patients with SEPSECS phenotypes report serious intellectual and developmental delays, spasticity, epilepsy, and axonal neuropathy. In addition, some patients suffer from autosomal recessive pontocerebellar hypoplasia type 2D (PCH2D), also known as progressive cerebellocerebral atrophy (PCCA), and have optic atrophy and hypotonia with progressive microcephaly caused by the atrophy process [38,39,40,41,42]. 

This review focuses on the implications of the selenoproteins named after letters of the alphabet, which are less known than the rest. These selenoproteins also play vital roles in the pathogenesis and prevention of many diseases (cardiovascular, gastrointestinal, hepatic, immuno-inflammatory, neurodegenerative, oncological, and muscular diseases, type 2 diabetes, etc.) as described below. The pathological conditions arise, as mentioned above, due to deficiency of selenium or cofactors, on the one hand, or the polymorphism in selenoproteins genes and synthesis, on the other hand.

An already large number of studies have shown that selenoproteins are involved in many processes in the organism, such as cellular oxidative stress, ER stress, antioxidant defense, and regulating the inflammatory and immune response [43,44,45,46], and have essential functions in antioxidant, anti-apoptosis, anti-inflammation, and other various complex mechanisms [47,48,49]. 

Various selenoproteins have an ER response function to ER stress conditions. ER is widely distributed in eukaryotic cells and is an essential organelle involved in protein processing and steroid synthesis [50]. When too many unfolded or misfolded proteins in RE are accumulated for a long period of time, it can lead to an imbalance in calcium homeostasis and, consequently, to an ER stress response, which, if it is not well managed, activates the corresponding signaling pathway and induces apoptosis [51]. The ER-resident selenoproteins involved in regulating ER stress include 15 kDa selenoproteins, DIO2 (iodothyronine deiodinase 2), SELENOS, SELENON, SELENOK, SELENOM, and SELENOT [52,53,54,55]. These ER-resident selenoproteins are implicated in ER stress, inflammation, and/or intracellular calcium homeostasis by regulating the calcium flux [56,57]. SELENON acts as a redox cofactor for ryanodine receptors (RyRs) [54], whereas Sep15, a redox enzyme, is associated with the proteins implicated in protein-folding quality control [58].

## 2. Implications of “Alphabet” Selenoproteins in the Pathology of Diseases

### 2.1. Implications of Selenoproteins in Cardiovascular Diseases

In the case of selenium deficiency conditions, vascular injury is triggered through multiple mechanisms, such as necrosis, apoptosis, and inflammation [50,59]. Increased selenoprotein expression in vascular endothelial cells may play a protective role by reducing abnormal cell adhesion induced by pro-inflammatory cytokines [60,61]. In addition, the downregulation of SELENOS can effectively prevent the development of cardiovascular diseases, such as atherosclerosis and hypertension [50]. Generally, selenoproteins protect the heart from the accumulated cholesterol in blood vessel walls by increasing the levels of coenzyme A in myocardial cells and increasing energy production [62].

Studies have shown that selenium deficiency could play an essential role in the pathogenesis of Keshan disease (KD), an endemic cardiomyopathy that leads to heart failure [63,64,65]. The disease was first reported in Keshan County in northeast China in 1935. Similar cases were reported in Nagano Prefecture in Japan and the northern mountains of North Korea in the 1950s. KD occurs because of low body selenium levels, a consequence of low selenium quantities in the soil in Keshan County [66,67], and oral selenium supplementation was found to eliminate Keshan disease a long time ago [68]. Regarding Keshan disease, an infection with Coxsackie virus B3 (CVB3) is a factor that also contributes to this disease [69,70], but the exact mechanism of selenium implication remains unclear [71].

Keshan disease is characterized by cardiac arrhythmia, acute heart failure, and congestive heart failure, and it is classified into acute, sub-acute, chronic, and latent KD. Nowadays, acute and sub-acute cases are almost absent. Only chronic and latent cases are reported, but rare, in many geographical areas. Besides KD, selenium deficiency is also correlated with other cardiovascular diseases, such as cardiomyopathies, atherosclerosis, coronary heart disease, myocardial infarction, and heart failure [43]. A recent study regarding the serum selenoprotein P and Keshan disease carried out in Heilongjiang Province in China concluded that the mean serum SELENOP levels of 28 KD endemic counties, meaning 56% of all surveyed endemic counties, were lower than those in all endemic counties and in a spatial regression analysis were positively correlated with the per capita GDP [72]. 

Besides Keshan disease, Chagas disease is caused by low selenium intake and a microbial parasite infection with Trypanosoma cruzi. Some patients infected with this parasite develop cardiomyopathy as a common cause of heart failure in South America [73]. Moreover, patients with Chagas disease tend to develop increased heart dysfunction, which may suggest a protective role of selenoproteins that remains to be fully elucidated [74,75]. 

SELENOT was shown to prevent free-radical injuries and the death of the cell during ischemia/reperfusion as SELENOT-derived peptides protect the heart from these processes by inhibiting apoptosis and oxidative stress [52]. In regulating cardiac apoptosis and survival mechanisms during cell stress conditions, ER stress has an essential role. The involvement of selenoproteins in regulating the mechanisms and pathways of the ER stress response represents only a component in that laborious and complex response in the organism. The ER stress induced by misfolded proteins is regulated by SELENOK in association with SELENOS, whereas SELENOM, SELENON, and Sep15 may regulate the cardiac response to ER stress [76]. It is well known already that SELENOK is an ER protein with an antioxidant function in cardiomyocytes, having a high mRNA expression in the heart [77].

Plasma SELENOP supplies cells with selenium, providing the necessary support for the optimal expression of selenoproteins. Moreover, SELENOP reduces peroxynitrite induced by protein oxidation and nitration and lipid and LDL peroxidation by oxidizing TRX (thioredoxin reductase) in return [78].

Clinical studies examining the correlation between selenium status and cardiovascular pathology mortality have provided contradictory data, but low selenium levels correlate with the risk of myocardial infarction [79,80]. 

Schomburg et al. reported a strong association between low SELENOP levels and the mortality risk associated with all causes, including cardiovascular mortality and a first cardiovascular event. The studies were performed on a large group of Swedish subjects with no history of cardiovascular events [81]. In addition, Schomburg et al. concluded the hypotheses of the mechanisms involved in the SELENOP influence on modifying cardiovascular risk [81,82]. These hypotheses are as follows: SELENOP transports selenium to tissues with specific uptake receptors ApoER2 or megalin, so selenoprotein biosynthesis increases to play roles in antioxidative defense and in regulating the protein quality-control systems. SELENOP is capable of catalyzing the degradation of phospholipid hydroperoxides by exhibiting GPx (glutathione peroxidase) activity, thereby protecting the cell membrane integrity [83] and LDL particles from oxidation [84]. SELENOP reduces peroxynitrite [77] and associates with the extracellular matrix via the heparin-binding domain [85]. SELENOP binds heavy metals, such as Cd, As, and Hg, preventing toxicity in the plasma [86] and reducing oxidative stress. Studies carried out refer to a cohort with Hg-exposed subjects and do not apply to the general population that is not exposed to Hg. A recent study has shown that subjects with high selenium intake and levels were less hypertensive and had reduced stroke and myocardial infarction than those with low selenium levels (Table 2) [87].

### 2.2. Implications of Selenoproteins in Liver Diseases

Many experiments have demonstrated that selenoproteins are involved in nonalcoholic fatty liver disease (NAFLD), which is, nowadays, considered the most common chronic liver disease and associated with serious complications, such as obesity and/or insulin resistance [132]. Studies found that the SELENOP levels were positively correlated with insulin resistance and NAFLD, but for serum selenium levels, the conclusions were different [88,132]. Wang et al. have shown that adding 1.0 mg/kg of Se can reduce the liver damage induced by high fat in a NAFLD pig model [89]. Moreover, Zhu et al. identified several upregulated selenoproteins in mild NAFLD liver samples compared to healthy controls, such as SELENON, SELENOP, SELENOT, SELENOW, DIO2, DIO3, GPx4, and GPx5, suggesting that in NAFLD, selenium-related processes are progressively perturbed [90]. In addition, other experiments revealed the essential role of selenoproteins in hepatic function after genetically excluding them in mice, which, under these conditions, developed hepatocellular degeneration and necrosis, leading to early death [133]. 

The liver secretory selenoprotein SELENOP is related to insulin resistance [134]. By administrating native selenoprotein P, the insulin signals are broken down to manage the insulin function in both hepatocytes and myocytes. In contrast, the knockdown and exclusion of SELENOP enhance common reactivity to insulin and enhance glucose tolerance in mice [134]. At the same time, a selective loss of so-called housekeeping selenoproteins SELENOP, SELENOF, DIO1, and TXNRD1 determined the upregulation of the genes involved in cholesterol biosynthesis and the downregulation of the genes that have roles in cholesterol metabolism and transport, suggesting the effect of these selenoproteins in favoring hypercholesterolemia [91].

In an article, Stergios A. Polyzos et al. concluded that the association between Se or SELENOP and insulin resistance, representing a principal pathogenic factor in NAFLD, remains inconclusive. Results of clinical studies are conflicting, except those performed in advanced liver diseases, such as cirrhosis or hepatocellular carcinoma, in which lower plasma selenium and SELENOP are consistent findings [135].

Other studies regarding SELENOS have shown that its mRNA level in the liver of pigs induced by high fat can be significantly increased, and its expression is negatively correlated with the apoptosis rate and the symptoms of nonalcoholic steatohepatitis, suggesting that this selenoprotein may be essential in the protection of the liver from high-fat-induced damage [89]. 

It is already known that dietary selenium deficiency can reduce liver selenase activity and, consequently, lead to oxidative stress and so, afterward, initiates oxidative stress-related signals [136,137]. In this situation, redox imbalance is induced by regulating selenoproteins at mRNA and protein levels by blocking the GSH system while enhancing GSH synthesis and catabolism [137]. In hepatocellular carcinoma, selenium plays an immunomodulatory role by regulating oxidative stress, inflammation, immune response, cell proliferation and growth, angiogenesis, signaling pathways, and apoptosis [136,138]. As shown by Sang et al., Se concentration was usually low in patients with hepatocellular carcinoma, and enhancing the Se concentration via exogenous supplementation was correlated with reducing the number and size of tumors [138].

Recent experiments have demonstrated that in the liver, there is a group of proteins called hepatokines, such as selenoprotein P, fetuin-A, and fibroblast growth factor-21 (FGF-21), that directly affect glucose and lipid metabolisms, similar to adipokines and myokines [139]. 

A serial analysis of gene expressions revealed that SELENOP is associated with insulin resistance in humans [140]. Studies have also shown that patients with type 2 diabetes mellitus and those with NAFLD have higher serum levels of this type of selenoprotein than healthy controls [141,142,143]. Moreover, it was found that salsalate and adiponectin ameliorated palmitate-induced insulin resistance in hepatocytes by inhibiting the selenoprotein P via the AMPK-Forkhead box protein O1α (FOXO1α) pathway, suggesting that this action might be a novel mechanism in mediating the antidiabetic effects of salsalate and adiponectin [143,144].

### 2.3. Implications of Selenoproteins in Intestinal Diseases

There is strong evidence that Se levels are linked to the incidence and severity of intestinal diseases, which have become very frequent and serious pathologies in the world, including inflammatory bowel disease (IBD) and colorectal cancer (CRC) [145,146]. Inflammatory bowel disease is a generalized term that includes Crohn’s disease (CD, regional ileitis) and other ulcerative colitis. Selenium reduces intestinal inflammation due to the action of selenoproteins, which have a protective role. In intestinal infections, their actions involve type-3 innate lymphocytes (ILC3) and T-helper cells 17 (Th17), which protect the intestinal barrier essential for maintaining physiological intestinal function [147,148]. Inflammation leads to barrier damage by increasing ROS (reactive oxygen species) production, while dietary Se supplementation can reduce their levels [148]. 

SELENOP is significantly reduced in the serum of Crohn’s disease (CD) subjects, and its serum concentration is negatively correlated with CRC risk [76].

Selenoprotein P originates from the colonic epithelium and represents the source of antioxidant-mediated cancer protection associated with colitis. In contrast, the downregulation promotes oxidative stress in ulcerative colitis [149]. Intestinal epithelial SELENOP knockdown increases the tumor load and genomic instability in cancer associated with the colitis model, suggesting its important role in the development of colon cancer [147,149]. Moreover, reduced selenium levels promote helper T-assisted 1 (Th1) cell differentiation in patients with Crohn’s disease. Selenium supplementation can inhibit Th1 cell differentiation through SELENOW, eliminate cytoplasmic ROS, and relieve symptoms in patients with Crohn’s disease [150]. In addition, experiments performed both in vitro and in vivo on Sep15 knockout colon cancer cells or mouse models have shown a reversal of the colon cancer phenotype and a reduction in the number of chemical-induced tumors [58,137].

SELENOS and SELENOK have also been implicated in inflammation and IBD [92,93,94]. It has been reported that increased production of cytokines has an inflammatory effect, with a decrease in the expression of SELENOS. Moreover, in the absence of SELENOK, the inflammatory cytokines decrease [93]. These findings must be further investigated.

In IBD, many immune cells, such as macrophages, T-cells, and innate lymphoid cells, are involved in this pathological condition, and studies have shown the important impact of selenium and selenoproteins in inflammatory signaling pathways implicated in the pathogenesis of this disease. Two transcription factors, nuclear factor-κB (NF-κB) and peroxisome proliferator-activated receptor γ (PPARγ), involved in activating immune cells and also implicated in various stages of inflammation, are impacted by the Se status. In addition, there is a correlation between the levels of NF-κB in the gut and the severity of IBD. Before resection surgery for Crohn’s disease, histological colon samples revealed a correlation between NF-κB levels and histological score, where higher levels led to a higher histological score [151]. Because NF-κB is a redox-sensitive transcription factor, it is also regulated by selenoproteins, which possibly act as antioxidants and can alleviate the symptoms of IBD [152]. Studies regarding SELENOP, which has both reductase and peroxidase activities, have shown that it is decreased in IBD. The oxidative stress developed during IBD can lead to the activation of NF-κB, so selenoproteins SELENOP and GPx2 (glutathione peroxidase 2) have the role and ability of reducing this stress, and this could lead to a decrease in the activation of NF-κB [153]. 

PPARγ is a key receptor that is highly expressed in the epithelial cells of the colon, second to adipose tissue, and, like NF-κB, has been implicated in the inflammation of the colon [154]. In contrast with NF-κB, whose expression is increased in IBD, in the case of PPARγ, a greater decrease is observed in patients suffering from ulcerative colitis compared to those suffering from Crohn’s disease [155]. 

Selenium plays an essential role in activating PPARγ and its ligands, which are derived from the arachidonic acid pathway of cyclooxygenase activity in macrophages. Selenium can increase both PPARγ and its ligand, the prostaglandin 15d-PGJ2 [93,156], so, eventually, under selenium supplementation, IBD would be significantly ameliorated.

Colorectal cancer (CRC) could be another complication of IBD, and patients suffering from IBD could have a high risk of developing CRC. Clinical trials that administrated Se supplements reported a decrease in the number of colorectal cancer cases compared to those patients that were administered a placebo [157]. Oxidative damage to DNA can lead to tumor development; in that case, selenoproteins can decrease the risk of CRC [157], so selenium and selenoproteins can be used as chemoprotective agents, since selenium is involved in regulating apoptosis and proliferation of the intestinal epithelium [153].

### 2.4. Implications of Selenoproteins in Cancer

As many studies have demonstrated, both selenium and selenoproteins play important roles in the occurrence of tumors and the progression of the malignant process [158,159,160,161]. 

Many selenoprotein gene polymorphisms have been linked to the risk of developing cancer. Polymorphisms in SELENOP, besides GPx2 and GPx4 (glutathione peroxidases), have been implicated in colorectal cancer [95,96]. Sep15 polymorphisms have been related to an increase in lung cancer risk [97]. SELENOS promoter polymorphisms have been linked to gastric cancer [98]. Recent experiments have shown that epistasis between the polymorphism of SELENOS and mitochondrial superoxide dismutase (SOD) has been linked to prostate cancer risk [162]. Moreover, changes in the expression of SELENOP, Sep15, GPx1, GPx2, and TrXR1 (thioredoxin reductase 1) have been related to different forms of cancer [161,163].

The downregulation of SELENOP, GPx1, and GPx3 is associated with tumorigenesis in colon cancer [147,149,164,165]. Sep15 is downregulated in liver, prostate, and lung cancers [166,167,168,169,170]. In contrast, the upregulation of Sep15 occurs in bladder tumors and bladder cancer cells [164]. 

SELENOK acts as a tumor suppressor in human choriocarcinoma cells because it negatively regulates human chorionic gonadotropin β subunit and β-HCG expression, which may be used as a novel therapeutic target for human choriocarcinoma in vitro [99]. In addition, regarding SELENOK, it was found that this selenoprotein is critical in promoting calcium fluxes that induce melanoma progression [100,101].

Numerous analyses were performed using NPC (Nutritional Prevention of Cancer) trials to determine whether selenium acts as a cancer-preventing agent. One of them has referred to the possibility that selenium supplementation could reduce the risk of skin carcinomas. The trials concluded that although the incidence of skin cancer did not differ between those groups from the trials, the total incidence of cancer decreased, including prostate, lung, and colorectal cancer [171]. The studies confirmed the protective effect of selenium supplementation in preventing prostate cancer [172]. Another recent study, the SELECT (Selenium and Vitamin E Cancer Prevention) study, found no significant decrease in prostate cancer after selenium supplementation. The SELECT study used purified selenomethionine, while the NPC study used selenized yeast [173].

### 2.5. Implications of Selenoproteins in Neurological Diseases

The brain retains selenium even under conditions of dietary selenium deficiency. Selenoproteins are most expressed in the brain, especially in cortex and hippocampus dysfunction [174,175]. Selenoproteins are essential for physiological brain function, and a decline in their function can lead to impaired cognitive function and neurological diseases [175,176,177,178]. ROS actions and damage are taking place in neurodegenerative disorders, such as Alzheimer’s disease (AD), Parkinson’s disease (PD), Huntington’s disease (HD), epilepsy, ischemic damage, brain tumors, exposure to environmental toxins, and drugs [178].

#### 2.5.1. Implications of Selenoproteins in Alzheimer’s Disease (AD)

Alzheimer’s disease, the most common type of progressive dementia, involves parts of the brain that control thought, memory, and language. AD manifests in memory loss, impaired cognitive function, and changes in behavior and personality [179]. The brains of AD patients accumulate abnormal amounts of extracellularly amyloid plaques consisting of the protein amyloid β, and tau proteins, as well as intracellularly as neurofibrillary tangles, form in the brain, affecting neuronal functioning and connectivity, resulting in the progressive loss of brain function. The abnormal interaction of β-amyloid 42 with copper, zinc, and iron induces peptide aggregation and oxidation in AD. Amyloid β degradation is mediated by extracellular metalloproteinases, neprilysin, insulin-degrading enzyme (IDE), and matrix metalloproteinases. In their autopsy studies, Dorothea Strozyk et al. found a strong inverse correlation between cerebrospinal fluid β-amyloid 42 and cerebrospinal metals, such as copper, zinc, iron, manganese, and chromium, with no association with selenium or aluminum. Moreover, it was also found a synergistic interaction of elevated copper and zinc with lower cerebrospinal fluid β-amyloid 42 levels [180].

Most cases of AD are late-onset and progress with age [181].

Studies have shown that several autosomal dominant mutations can result in early-onset AD. One of these mutations is in presenilin-2, an enzyme involved in processing amyloid precursor protein [182].

It is believed that SELENOM might play a suppressive or protective role in AD because, in a mouse model that overexpressed the human mutation in presenilin-2, the levels of brain SELENOM were reduced [102].

The overexpression of SELENOM, as well as selenium supplementation and treatments, activates ERK signaling, leading to a decrease in tau phosphorylation, α-secretase, and γ-secretase activities and an increase in β-secretase activity [183]. In mice, SELENOM overexpression due to selenium treatment led to significant ROS inhibition, reduced mitochondrial damage, and decreased γ-secretase activity [184]. The enzyme γ-secretase is a multimeric protease complex composed of presenilin and four additional cofactors, nicastrin, Aph-1, Pen-2, and TMP-21 [185,186].

Another selenoprotein, SELENOP, is abundant in the human brain in neurons and ependymal cells [187]. SELENOP expression in the brain increases with age, suggesting a probable role of SELENOP in decreasing oxidative stress [188]. Studies found that the genetic deletion of SELENOP results in a decrease in central-nervous-system-associated selenium levels, suggesting that other selenoproteins compensate for the SELENOP deficiency and that the basal brain selenium levels probably consist of a priority for the available selenium in the body [181,189]. Selenoprotein P deficiency determines subtle spatial acquisition learning and memory deficits and severely disrupts synaptic plasticity in area CA1 of the hippocampus. Researchers concluded that it is difficult to discern whether these effects are due to SELENOP or the loss of the selenium transport to the brain [190].

Bellinger et al. investigated the expression of SELENOP in the post-mortem human brain and discovered a unique expression pattern of SELENOP within the center of neuritic (dense-core) plaques and found a co-localization of SELENOP with plaques and neurofibrillary tangles, which suggests a possible role of SELENOP in reducing the oxidation accompanying plaques [191].

SELENOP is highly influenced by dietary selenium, so selenium supplementation may play a direct neuroprotective role by increasing SELENOP expression [192]. Several studies have even suggested that selenium supplementation can decrease amyloid toxicity in cell cultures and animal models [180,193].

Considering oxidative stress, a hallmark of Alzheimer’s disease, SELENOP, due to its prominent antioxidant role, might act in AD by protecting neurons against oxidative lesion damage or transporting selenium so that other antioxidant selenoproteins can be further synthesized. SELENOP encodes two His-rich regions that are high-affinity binding sites for transition metals, suggesting its possible role in blocking metal-mediated β-amyloid 42 aggregation and further subsequent ROS (oxidative reactive species) generation [103]. In addition, studies found that SELENOP inhibits tau aggregation due to its two His-rich domains and disassembles formed aggregates of tau that are induced by the presence of Cu^+^/Cu^2+^ [194]. These two His-rich regions of SELENOP associate with the acidic tail of α-tubulin via an ionic interaction, suggesting that SELENOP can possibly be involved in microtubule events that are associated with the maintenance of cell polarity, intracellular transport, and cell division and migration [175,195].

#### 2.5.2. Implications of Selenoproteins in Parkinson’s Disease (PD)

Parkinson’s disease (PD) is a neurodegenerative disorder characterized by a loss of motor control, caused mainly by a dramatic loss of dopaminergic neurons in the midbrain substantia nigra [196,197]. Before cell loss, Lewy bodies are formed, which are intracellular bodies of insoluble protein aggregates of ubiquitinated α-synuclein [198]. Symptoms of PD include rigidity, bradykinesia, resting tremor, flexed posture, “freezing”, loss of movement control, and postural reflexes, with mood changes and cognitive impairments occurring in the later stages of the disease. Parkinson’s disease is the major cause of Parkinsonism, a clinical syndrome comprising combinations of motor problems as mentioned above [196]. 

The substantia nigra and putamen have higher selenium concentrations than other brain regions [149]. Selenium may play an important role in PD by reducing oxidative stress via selenoproteins [149]. In PD, it was found that plasma selenium decreases [199]. An explanation might be that there is an intense selenium utilization for selenoprotein synthesis in the brain, possibly to prevent further oxidative damage. SELENOP is found together with presynaptic terminals in the striatum. Besides SELENOP, GPx4 is also decreased in the substantia nigra in patients with PD [104]. Moreover, glutathione levels in the midbrain are also decreased before clinical symptoms, so GPx function is impaired, promoting oxidation [200,201].

Loubna Boukhzar et al. found that SELENOT plays a major role in protecting dopaminergic neurons against oxidative stress because, according to their studies, its loss enhanced the neurotoxin-induced degeneration of the nigrostriatal system, decreased dopamine secretion, and impaired motor function. These studies represent the first data that demonstrated that SELENOT is involved in the nigrostriatal pathway and the involvement of a selenoprotein in maintaining the functionality of the dopaminergic system and preserving motor function under oxidative stress conditions [105]. Previous studies have shown that only several selenoproteins, particularly TrxR (thioredoxin reductase), can protect neuronal cells [202,203]. SELENOT exerts an oxidoreductase activity like TxrR through its thioredoxin-like fold, so it represents a new important component of the thioredoxin system, localized in ER, in addition to the cytosol and mitochondrial TrxR1 and TrxR2 [204]. Experiments performed using quantitative PCR, immunochemical, and Western blot analyses revealed that SELENOT expression is significantly increased in PD mice models, both in vitro and in vivo. The researchers concluded that SELENOT acts as a gatekeeper of redox homeostasis in the nigrostriatal pathway that is essential for physiological dopamine secretion and, therefore, maintaining motor function under oxidative stress conditions. Moreover, the oxidoreductase activity in the nigrostriatal pathway from the substantia nigra pars compacta to the caudate putamen prevents rapid-onset motor impairments in mouse models of PD [105]. Alongside Boukhzar et al., Bellinger et al. reported an altered expression of SELENOP and GPx4 in survival nigral cells and dystrophic putamen dopaminergic fibers in Parkinson’s disease patients, suggesting that different selenoproteins may be useful as complementary biomarkers of PD [105].

#### 2.5.3. Implications of Selenoproteins in Epilepsy

Epilepsy is a chronic neurological disease characterized by periodic episodes of abnormal electrical activity (seizures) that cause temporary interruptions in normal brain function. The types of seizures vary and are clinically classified into partial epilepsy syndromes with a specific location and generalized epilepsy syndromes that spread throughout the brain [205]. Generalized syndrome seizures typically originate simultaneously in both cerebral hemispheres, whereas in partial ones, seizures originate in one or more foci but can spread throughout the brain. Epilepsies are also classified according to their etiology as idiopathic and symptomatic. Idiopathic forms develop from reappearing unprovoked seizures, have no apparent neurological problems, have an unknown cause, and may be influenced by genetic factors. Symptomatic epilepsies are sporadic and characterized by multiple seizures, and they have many causes, such as cellular and anatomical inborn brain abnormalities and impaired metabolic brain processes [206]. 

A considerable number of studies have demonstrated an inverse correlation between serum selenium levels and epileptic seizures [207,208]. In infants, studies have also shown that low selenium serum levels lead to seizures and neurological disturbances [208]. Also, even in the case of febrile seizures, which are not abnormal in childhood, there is an inverse correlation with serum selenium levels, suggesting the preventive role of selenium against certain types of epilepsy [209]. In addition, selenium deficiency promotes the risk of seizures in childhood epilepsy [207,210,211]. However, a recent study demonstrated decreased serum selenium and zinc in patients with idiopathic intractable epilepsy, independent of the nutrition intake [212]. It is mentioned that epilepsy may increase the utilization of selenium even if the intake is adequate, probably supporting the activity of GPx antioxidant activity and other selenoproteins to prevent the cytotoxicity of seizures. This hypothesis is confirmed by the increased expression of SELENOW, GPx1, and TrxR1 observed in the excised brain tissue of patients with severe epilepsy requiring surgery [106].

Epilepsy, ischemia, and brain trauma may trigger the initiation of a cascade of free radicals and the activation of pro-apoptotic transcription factors with consequent neuronal loss [213].

The knockout of SELENOP increases seizures in selenium deficiency, while brain-specific knockout of all selenoproteins leads to severe seizures [107].

### 2.6. Implications of Selenoproteins in Muscle Diseases

Selenium deficiency causes muscle disorders, observed in both humans and animals, especially in regions with low selenium soil quantities. Selenium deficiency causes myotonic dystrophy with weakness and muscle pain. White muscle disease (WMD) is a muscle disorder developed in farmed regions, with livestock raised on land with low selenium levels [214]. The muscles of affected animals appear paler than normal and may show distinct longitudinal striations or a distinct chalky appearance due to abnormal calcium storage. This disease can affect both skeletal and cardiac muscles where SELENOW is highly expressed. SELENOW was the first selenoprotein described to be linked to a muscular disorder [108]. SELENOW is less abundant in the muscles of WMD animals. The sarcoplasmic reticulum of the muscles in WMD has impaired calcium sequestration, resulting in the calcification of skeletal and cardiac muscle tissue. Studies have also revealed that SELENOW is complexed with glutathione in the cytosol through a covalent linkage to one of the cysteine residues. SELENOW is named after white muscle disease, and its levels are upregulated in response to exogenous oxidants in muscle cells [109,110]. 

The term “muscular dystrophy” includes several muscular disorders characterized by the slow degeneration of muscle tissue [111].

Several of these muscular disorders have genetic causes. One of these muscular dystrophies, termed “Multi-minicore disease”, is a recessively inherited form characterized by multiple small lesions and cores scattered throughout the muscle fiber on muscle biopsy and clinical features of a congenital myopathy [112]. Although there is genetic heterogeneity with clinical variability, the classic phenotype is easily recognizable via spinal rigidity, early scoliosis, and respiratory impairment. Multi-minicore disease occurs due to recessive mutations in the selenoprotein N gene (SEPN 1), whereas recessive mutations in the skeletal muscle ryanodine receptor gene (RYR 1) have been associated with wider clinical features, such as ophthalmoplegia, distal weakness, and wasting or predominant hip girdle involvement, resembling central core disease (CCD). In CCD, there may be a histopathologic continuum at biopsy, with multiple larger lesions (“multicores”) due to dominant RYR 1 mutations [112,113]. The role of SELENON in these diseases remains elusive because its exact function is still incompletely known. One mutation causing Multi-minicore Disease involves the loss of a selenium-response element (SRE), a cis element found in some selenoproteins and the SECIS element. The SRE is localized within the RNA-coding region following the UGA codon. An SRE mutation prevents read-through, leading to an early termination of translation [114].

Ryanodine receptors are channels in the sarcoplasmic reticulum responsible for the redox-sensitive calcium-stimulated release of calcium from intracellular stores [215]. These receptors potentiate calcium signals that may be initiated from the membrane calcium channels and receptors or via other calcium store channels, for example, InsP3-sensitive channels [216]. 

All the early-onset muscular disorders caused by mutations in SEPN 1 gene include, besides multi-minicore myopathy (MmD) [217,218], congenital muscular dystrophy with spinal rigidity (RSMD1) [124,219,220,221], rare cases of desmin-related myopathy with Mallory body-like inclusions (MB-DRM) [222], and congenital fiber-type disproportion myopathy (CFTD) [223].

Regarding glucose tolerance in muscles, adenosine monophosphate-activated protein kinase (AMPK) is a mediator in the regulatory activity of SELENOP, so this fact considers SELENOP a future therapeutic target in diabetes mellitus 2 types [224].

### 2.7. Implications of Selenoproteins in Inflammation and Immune Response

SELENOK is one of the selenoproteins that are essentially involved in calcium flux, T-cell proliferation, and neutrophil migration in immune cells, also protecting the cells from ER-stress-induced apoptosis [91]. In regulating immunity, SELENOK represents a cofactor of enzymes involved in the key post-translational transformations of proteins by enhancing the catalytic efficiency, and it also has a biochemical role through antioxidant and protein repair [225]. 

Selenoprotein S is also involved in the immune response. SELENOS is an ER membrane protein that interacts with the ER membrane protein Derlin and the VCP (p97, valosin-containing protein), which is a cytosolic ATPase [226,227,228]. VCP is translocated to the ER membrane by binding to SELENOS during endoplasmic-reticulum-associated degradation (ERAD) and is responsible for the retro-translocation of misfolded proteins from the ER, where they are tagged with ubiquitin and then transported to the cell proteasome [181,226]. Because of its action, SELENOS is also named VIMP for VCP (valosin-containing protein)-interacting membrane protein [115]. 

Selenoprotein K is another known p97(VCP)-binding-selenoprotein, and the expression of both SELENOK and SELENOS is increased under ER stress. The translocation of p97 (VCP) to the ER membrane is regulated by SELENOS, not by SELENOK, but p97(VCP) is required for associating SELENOK with SELENOS. In addition, the interaction between p97(VCP) and SELENOK is regulated via SELENOS. The degradation of ERAD substrates requires p97 (VCP), and its translocation from the cytosol to the ER membrane is essential to shuttle ERAD substrates to the proteasome. SELENOK and SELENOS are essential to forming the ERAD complex, alongside p97(VCP), in their response to ER stress [116,117].

The polymorphisms of the SELENOS promoter can lead to the downregulated expression of SELENOS and cause the accumulation of many misfolded proteins in ER. Subsequently, ER stress can induce NF-κB, which can upregulate inflammatory cytokines and lead to apoptosis [181].

The expression of SELENOS in liver cells is regulated by inflammatory cytokines and extracellular glucose [229,230]. Studies reveal that polymorphisms significantly impair the expression of selenoprotein S, for example, a change from G to A at position –105 in the SELENOS promoter [231]. Moreover, subjects with this polymorphism have increased plasma levels of inflammatory cytokines TNFα and IL-1β, and this polymorphism is correlated with increased incidence of stroke in women [232], pre-eclampsia [233], coronary heart disease [234], and gastric cancer [103]. The -105 polymorphism exhibits epistasis with the -511 polymorphism of IL-1β, and both increase the risk of rheumatoid arthritis, although there was no correlation of polymorphisms with rheumatoid arthritis alone [235]. On the other hand, other studies did not find correlations with stroke [236], autoimmune disorders [237], or inflammatory bowel disease [112].

Selenoproteins are also implicated during wound healing. SELENOS, SELENOP, GPx1, and GPx4 perform various actions in the inflammatory phase, such as antioxidant actions, the inhibition of inflammatory cytokines, and the elimination of peroxynitrite radical ion [118,119,120]. Wound healing is a complex process following many cascades of events that have different stages, such as hemostasis (1), inflammation (2), proliferation (3), and remodeling or maturation (4) [14]. 

In the inflammatory phase of wound healing, soluble factors are released, such as chemokines and cytokines, to phagocyte the debris, bacteria, and damaged tissues. Recent studies have revealed that SELENOS has an essential role in this inflammatory phase. As mentioned in this review, SELENOS is a transmembrane protein found in ER with a function that includes removing the misfolded proteins from the ER lumen, protecting the cells from oxidative damage, and contributing to ER-stress-induced apoptosis. The depletion of SELENOS by siRNA increases the release of inflammatory cytokines IL-6 and TNF-α, so SELENOS may regulate the cytokine production in macrophages and subsequently participate in controlling the inflammatory responses [14].

Other research has shown that the results of a real-time PCR study revealed a lower expression of SELENOP mRNA in whole blood in Kashin–Beck Disease (KBD) patients compared to healthy controls, with a higher expression in the articular cartilage tissue. These findings have suggested that the decreased SELENOP mRNA expression in KBD reflected the selenium deficiency condition in KBD patients. Under the selenium deficiency condition, the glutathione (GSH) metabolism is impaired and glutathione peroxidase activity decreases, leading to an increase in oxidative damage in bone and articular cells [121]. KBD is a particular type of chronic osteoarthritis, an endemic disease in the northern part of China, Russia, and a few northern areas of North Korea. KBD mainly affects the knee, ankle, and hand joints, causing articular cartilage damage and chondrocyte apoptosis. KBD has traditionally been classified as a non-inflammatory osteoarthritis, but recent studies demonstrate that inflammation plays an important role in its development and evolution. Recently, it was found that KBD is not only an endemic disease anymore because of non-endemic factors such as age, altered biomechanics, joint trauma, and secondary osteoarthritis that also can cause this disease. It was concluded that advanced stages of joint complications and failure in KBD are tightly linked with the immune response, and the subsequent stage of chronic inflammation leads to the progression of the disease [238].

### 2.8. Implications of Selenoproteins in Type 2 Diabetes Mellitus

SELENOP, which originates from the liver, is essential for supplying extrahepatic tissues with the selenium required for the biosynthesis of selenoproteins. It has been shown that increased plasma SELENOP levels are associated with hyperglycemia in patients with type 2 diabetes mellitus (T2DM) [122,123]. Moreover, it was recently found that high SELENOP plasma levels are also associated with hepatic steatosis and fibrosis in NAFLD patients [88]. Insulin sensitivity in the liver and skeletal muscle was improved in SELENOP-deficient mice, while intraperitoneal injection with SELENOP impaired insulin signaling, suggesting that SELENOP is a hepatokine capable of inducing insulin resistance [101,210].

Several studies have revealed that in humans, plasma SELENOP levels were saturated at a daily intake of approx. 50–100 μg Se and did not further increase by ingesting selenium supplements in larger doses [101,210,239]. High plasma levels may be an accompanying effect of insulin resistance and hyperglycemia because research has shown that its hepatic biosynthesis is suppressed by insulin and increased by high glucose concentrations [101,240,241]. Selenoprotein P hepatic transcription is regulated similarly to that of a gluconeogenic enzyme through transcription factors FoxO1 and HNF-4α together with the co-activator PGC-1α and may also become dysregulated in hyperglycemia and insulin-resistance states [101,241,242]. 

Many researchers suggest that suppressing SELENOP may provide therapeutic ways to treat T2DM and its vascular complications [243]. Metformin (an antidiabetic drug) phosphorylates and inactivates FoxO3a via the activation of AMPK (AMP-activated protein kinase) and suppresses SELENOP expression in hepatocytes [244]. Eicosapentaenoic acid (an ω-3 polyunsaturated fatty acid) downregulates SELENOP by inactivating sterol-regulatory-element-binding protein-1c, independently of the AMPK (AMP-activated protein kinase) pathway [245]. Moreover, the novel molecular strategy for neutralizing SELENOP monoclonal antibody AE2 reportedly improved glucose tolerance, insulin secretion, and insulin resistance in vivo and in vitro [246].

Serum SELENOS, mostly secreted by hepatocytes, was associated with T2DM and its macrovascular complications (macroangiopathy) [125,247]. SELENOS has antioxidant and anti-inflammatory functions, so it contributes to maintaining the morphology of ER and the regulation of ER stress, suggesting that it may be involved in the occurrence and development of T2DM [248,249]. Moreover, several genetic polymorphisms in the SELENOS gene were demonstrated to be related to T2DM, serum insulin levels, blood glucose levels, and the homeostasis model assessment of insulin resistance [247,250].

SELENOK protects cells from apoptosis induced by ER stress and is essential for promoting Ca^2+^ flux during immune cell activation [19,251]. Experiments performed in vitro have shown that the expression of SELENOK, as well as DIO2 (deiodinase2), was downregulated by about 10% due to high glucose levels [126].

Recent studies have discovered the role of SELENOV in protection against the oxidative damage of oxygen and nitrogen reactive species (ROS/RNS) mediated by ER stress [252,253].

### 2.9. Implications of Selenoproteins in Obesity

Adipocyte SELENOP is significantly influenced by pro-inflammatory stimuli involved in the pathogenesis of obesity and its associated metabolic disorders. Studies have shown that differentiated adipocytes responded to omentin exposure in vivo with a significant decrease in SELENOP expression and the pro-inflammatory response [127]. Omentin is a novel adipokine with insulin-sensitizing effects and is especially produced by visceral adipose tissue, where circulating levels are decreased in insulin-resistant conditions, such as obesity and diabetes. Other studies concluded that SELENOP gene expression in 3T3-L1 adipocytes was reduced in response to TNF-α or H2O2 treatment, indicating a link between adipose tissue inflammation and oxidative stress in obesity and altered selenoprotein metabolism [128]. Moreover, the negative regulation of SELENOP levels occurs in increased pro-inflammatory cytokine IL-6 and MCP1 induced by hypoxia [128].

Researchers have demonstrated a significant decrease in SELENOP gene expression in the adipose tissue of obese (ob/ob), HFD-fed, Zucker rats, and insulin-resistant patients [254]. When leptin treatment was administrated in ob/ob mice, there was a shift to lipid catabolism genes that involved the inhibition of SREBP1 (sterol regulatory element-binding protein 1) downstream signaling, as well as the upregulation of SELENOP and SREBP1 expression in the liver [255]. In contrast, its expression was found to be two-fold higher in the obese adipose tissue of OLETF rats [256].

SELENOS expression in adipose tissue is increased in obese patients and is significantly correlated with anthropometric measures of obesity and insulin resistance. Studies performed in vitro using isolated human adipocytes have demonstrated that insulin upregulates its expression, suggesting a link between insulin resistance and SELENOS expression in obesity [129].

In the development of obesity and/or its associated metabolic impairments, methionine sulfoxide reductases (Msrs) may also be involved. Experiments studying diet-induced obesity in HFD-fed subjects, with 45% calories from fat, reduced both MsrA and MsrB (predominantly MsrB1, also known as SELENOR) activities and also their protein abundance in VAT (visceral adipose tissue) but not in SAT (subcutaneous adipose tissue) [130].

It has also been demonstrated that obesity upregulates the hepatic expressions of MsrB1, SELENON, SELENOP, and SELENOW, as well as GPx4 in diabetic patients by 33–35% compared to non-obese subjects [131].

## 3. Conclusions

Members of the selenoproteins family named after letters of the alphabet require, like other selenoproteins, a common set of cofactors for their synthesis, depending on dietary selenium intake. The energy consumed for their synthesis suggests their great importance for cell physiological function, a consequence of their quite varied roles. “Alphabet” selenoproteins are also involved in numerous diseases and pathological conditions, including type 2 diabetes, cardiovascular, muscular, brain, liver, neurodegenerative, immuno-inflammatory, and gastrointestinal diseases, as described above in this article. Consequently, it is also of great importance to expose the medical correlations and implications of these “alphabet” selenoproteins, which are less known than the rest of the selenoproteins, which, otherwise, could risk remaining overlooked, especially regarding establishing quicker prevention, on one hand, and the diagnostic and therapeutical management of the diseases, on the other hand. Given the numerous and varied roles of these selenoproteins, strategies to target the expression of specific selenoproteins could and should be considered in the future for therapeutic and prevention management. Although the functions of several selenoproteins remain unknown, further research and understanding of each member of this whole selenoproteins family, including the “alphabet” selenoproteins, will be essential in establishing the health benefits of selenium.

## Figures and Tables

**Table 1:** The functions of “alphabet” selenoproteins.

“Alphabet” Selenoproteins	Functions	References
F (Sep15)	Protein (glycoprotein) folding	[3]
H	Redox homeostasis Control of cell cycle	[4,5]
I	Biosynthesis of phospholipids	[6,7]
K	Antioxidant Immune response Calcium-dependent signal transmissions	[8,9]
M	Antioxidant Calcium homeostasis Hypothalamic leptin signaling	[10,11,12]
N	Redox signaling Muscle development Calcium homeostasis	[13,14]
O	Redox function (possible)	[15]
P (Sepp1)	Selenium transportation Antioxidant	[16,17]
R (MrsB1)	Antioxidant Protein repair Methionine metabolism	[18]
S	Regulate inflammatory response Removes misfolded proteins in ER Induce ER stress apoptosis	[19,20,21]
T	Redox function Hormone synthesis Calcium mobilization	[22,23,24]
V	Specific expression in testes	[7]
W	Antioxidant	[25]

**Table 2:** “Alphabet” selenoproteins related diseases.

Related Disorders/ Diseases	Selenoproteins Involved
***Cardiovascular***	T [52], K in association with S, M, N, F (sep15) [60,61,62], P [77]
Keshan disease	P [69,70,71]
***Liver***	
NAFLD	P, N, T, W, S [88,89,90]
Hypercholesterolemia	P, F (sep15) [91]
***Intestinal***	
Crohn’s disease and	P [45]
colorectal cancer (CRC)	
Inflammation (IBD)	S, K [92,93,94]
***Cancer***	
Colorectal cancer (CRC)	P [95,96]
Lung cancer	F (sep15) [97]
Gastric	S [98]
Tumor suppressor in choriocarcinoma cells	K [99]
Melanoma progression	K [100,101]
***Neurological***	
Alzheimer’s disease (AD)	M [102], P [103]
Parkinson’s disease (PD)	P [104,105], T [105]
Epilepsy	W [106], P [107]
***Muscular***	
White muscle disease (WMD)	W [108,109,110,111]
Multi-minicore disease (MmC)	N [112,113,114]
***Immune response***	S [115], K [116,117]
Wound healing	S, P [118,119,120]
Kashin–Beck disease (KBD)	P [121]
***Type 2 Diabetes Mellitus***	P [122,123], S [124,125], K [126]
***Obesity***	P [127,128], S [129], R [130], N, W [131]

## Data Availability

Not applicable.
